# Six weeks of either EPA-rich or DHA-rich Omega-3 supplementation alters submaximal exercise physiology in endurance trained male amateurs

**DOI:** 10.3389/fnut.2025.1588421

**Published:** 2025-08-26

**Authors:** Andrew Blannin, George Boulton, Frank Thielecke

**Affiliations:** ^1^School of Sport, Exercise and Rehabilitation Sciences, University of Birmingham, Birmingham, United Kingdom; ^2^Department of Health Promotion, Swiss Distance University of Applied Sciences, Brig, Switzerland; ^3^T2 Bene Ltd., Allschwil, Switzerland

**Keywords:** Omega-3 index, EPA, DHA, submaximal exercise, heart rate, performance

## Abstract

**Purpose:**

Supplementation with Omega-3 fatty acids such as Docosahexaenoic acid (DHA) and/or Eicosapentaenoic acid (EPA) have been shown to lower submaximal exercise heart rate (HR) and whole-body oxygen consumption along with other positive exercise physiology adaptations. However, the impact of supplementation on exercise physiology is inconsistent. This could be due to existing study heterogeneity, including inconsistent use of EPA or DHA supplements. The current study aimed to investigate if EPA-rich or DHA-rich supplements are equally efficient at modifying physiological responses to submaximal exercise and potentially improving performance.

**Methods:**

Fifty-five endurance trained amateurs participated in a submaximal exercise test followed by a 24 km time trial (TT) before and after a six-week supplementation period. Participants were supplemented with either 3 g/day EPA-rich fish oil, DHA-rich algae oil, or a coconut oil placebo. Omega-3 index, submaximal exercising HR, rating of perceived exertion (RPE), respiratory exchange ratio (RER), and TT performance were all assessed.

**Results:**

The EPA-rich and DHA-rich supplements significantly increased the Omega-3 index, whereas the placebo supplement had no effect. Statistically significant changes between pre-and post-supplementation were found in submaximal exercise physiology. Both EPA-rich and DHA-rich supplementation lowered submaximal exercising HR (∆ = −4, *p* = 0.005) (∆ = −9, *p* ≤ 0.001) and RPE (∆ = −0.7, *p* ≤ 0.001) (∆ = −0.9, *p* ≤ 0.001), while only EPA-rich supplementation increased RER (∆ = +0.03, *p* ≤ 0.001). Change in Omega-3 index inversely correlated with both change in submaximal exercising HR (RHO = −0.43, *p* = 0.007) and RPE (RHO = −0.40, *p* = 0.013). TT performance improved in all three conditions, but there were no significant differences in the gains across the three conditions.

**Conclusion:**

This study adds further evidence that both EPA and DHA can alter submaximal exercise physiology, but further research is required to determine their effects on exercise performance outcomes.

## Introduction

Fine margins in sport require athletes to maximally optimize performance, with 11–100% of athletes using dietary supplements (1). It has been suggested Omega-3 fatty acids may enhance endurance exercise adaptations such as resting HR, exercise efficiency & increase V̇O_2_ max (2, 3). Several studies have reported decreased exercising HR and reduced whole-body oxygen consumption during submaximal exercise, leading to the suggestion of an improved exercise efficiency; for a review of these studies see Thielecke and Blannin 2020 (4). Despite these enhanced endurance adaptations, research into whether Omega-3 fatty acid supplementation adaptations improve endurance performance is still inconclusive and could be a result of large amounts of study heterogeneity such as differences in: dose, supplementation duration, EPA: DHA ratio, training status, Omega-6 in placebo, measure of bioavailability, and performance measure (time trial (TT) vs. time to exhaustion). A recent review by Anthony et al. (5) also supports a more consistent approach for future research in this field.

An early study of the effect of Omega-3 supplementation on endurance performance in trained cyclists showed no difference in a TT of around 1 hour (6). However, the supplementation period was very short at 3 weeks; perhaps not long enough for changes to develop (5). Furthermore no fatty acid profile was obtained of participants’ red blood cells (RBCs) to confirm if incorporation of Omega-3 fatty acids occurred (5). It is thought that for cardiovascular disease related outcomes the Omega-3 index needs to be above 8% in humans (7). Given that absorption and metabolism of a given dose of Omega-3 fatty acids may differ substantially in people, correlating the Omega-3 index with the outcome measures may be a better approach than using the amount of Omega-3 fatty acids consumed. While the Omega-3 index is a useful biomarker it underestimates the Omega-3 incorporation in target tissues such as cardiac and skeletal muscle. Von Schacky et al. (8) reported that only 1 of 106 German winter elite athletes had an Omega-3 index of ≥8%, suggesting a widespread deficiency and the potential need for increased Omega-3 intake among athletes. However most studies do not report Omega-3 index, but those that do often show only modest improvements, for example Hingley et al. (9) increased athletes Omega-3 index from 4.7 to 6.3%. In contrast, adopting a 12 week dosing strategy of 2,234 mg of EPA and 916 mg of DHA daily resulted in an increased Omega-3 index from 5.8 to 11.6% in endurance trained runners (10). The authors of that study also found supplementation increased V̇O_2_ max & running economy, but no change was found in 1500 meter running performance, which might be too short to reveal any difference.

Many studies have opted to use fish oil as the source of Omega-3 fatty acids in randomized control trials. However, the type of fish oil chosen can alter the EPA and DHA ratio. For example, some studies have used DHA-rich fish oil (2.4 g DHA & 0.8 g EPA) (2) while others have used EPA-rich fish oil (5 g EPA & 1 g DHA) (11). Some of the inconsistent effects of Omega-3 s on exercise performance might be due to the different ratios of DHA and EPA in the supplements provided across studies (4). Separate studies provide evidence suggesting DHA-rich supplements alter cardiac function (12), whereas EPA-rich supplements might alter oxygen delivery (13). In general, there is a need for human intervention studies to determine the independent effects of EPA and DHA on relevant outcomes (14). Directly comparing EPA-rich and DHA-rich supplementation protocols in a single study would begin to explore if they have similar benefits or not.

Another important consideration is the choice of placebo. While Omega-3 and Omega-6 fatty acids compete for certain enzymes in their metabolism, it seems advisable to use a placebo that contains no Omega-3 or Omega-6 fatty acids (such as coconut oil) (15), thereby reducing the risk of potentially altering Omega-3: Omega-6 absorption and/or incorporation into cell membranes.

Eight weeks of DHA-rich fish oil supplementation reduced HR and whole-body oxygen consumption during submaximal cycling (2), however, this had no impact on endurance performance, assessed using a time to exhaustion protocol at 55%-Watt max (2). Time to exhaustion protocols can be used as a performance indicator, although very few sports involve competing to exhaustion and exercising at one constant intensity (16). A more ecologically valid measure would be a TT as these have lower test to retest variation and mimic endurance performance more realistically (17).

The current study aimed to investigate the differences between supplementation with EPA-rich fish oil, or DHA-rich algae oil versus a true placebo (coconut oil) on the Omega-3 index, submaximal exercise responses, such as heart rate (HR), rate of perceived exertion (RPE), respiratory exchange ratio (RER) and a time trial (TT) performance test. EPA-rich oil consisted of 1.8 g of EPA and 1.2 g of DHA per day, while DHA-rich oil consisted of 2 g of DHA and 1 g of EPA per day. The main objective was to compare the supplement-induced changes in the submaximal exercise responses. It was hypothesized that 6 weeks of supplementation would increase the Omega-3 index in the DHA-rich algae oil and EPA-rich fish oil conditions, but not the placebo. Furthermore, it was expected that submaximal exercise HR would decrease in the DHA-rich group only. It was also hypothesized the two experimental conditions would provide significantly greater TT performance gains compared to the placebo group.

## Methods

### Participants

Sixty-nine endurance trained male amateurs from the disciplines of cycling, swimming, running, rowing, and team sports were recruited to participate in this study. In this early-phase study where establishing a clear cause-and-effect relationship was crucial, we focused only on male participants. Participants had to be endurance trained (3 regular endurance training sessions per week for the last year), 18–50 years old, healthy (as assessed by the general health questionnaire), male and a non-smoker. Participants were excluded before the trial if they: were participating in another clinical trial, were performing at international level in their discipline, had a recent or recurring injury, had an allergy from the following list (seafood, coconut oil or gelatine), regularly consumed more than two portions of oily fish a week or had been taking Omega-3 supplements (3 or more times a week for the last 3 months). Participants were asked to maintain their habitual exercise routine and diet throughout the study intervention period. Written informed consent was obtained from all participants and the experimental protocol was approved by the local research ethics committee (EX251019-1). Data were removed from the study where 14 participants did not complete the study (3), were injured (2), became ill (2) exhibited a lack of effort (2) or supplementation compliance was below 70% (5). Therefore 55 participants’ data are reported for submaximal and TT data. Participant characteristics are shown in Table 1.

**Table 1:** Participant characteristics.

Condition	Age (years)	Height (m)	Weight (kg)	BMI (kg/m^2^)	Predicted V̇O_2_max (ml/kg/min)
EPA-rich (fish oil) *N* = 18	22.9 ± 2.0	1.81 ± 0.01	76.0 ± 1.9	23.2 ± 1.9	49.8 ± 11.8
Control (coconut oil) *N* = 17	21.5 ± 1.0	1.79 ± 0.01	76.0 ± 1.6	23.8 ± 2.0	47.2 ± 6.9
DHA-rich (algae oli) *N* = 20	27.4 ± 2.7	1.80 ± 0.01	76.2 ± 1.4	23.6 ± 1.7	49.3 ± 9.0

### Research design

The study was performed as a double blinded, block randomized parallel control trial. Familiarization, baseline, and post-intervention tests were performed identically. Once each participant had been familiarized and screened to the lab protocol, participants then returned within the next month to complete their baseline measurements. Participants were then matched in groups of three based on: their sporting discipline, body composition and predicted V̇O_2_ max. They were then randomly assigned to Condition by a researcher external to the study. Each participant then consumed 3 g/day of either EPA-rich fish oil, DHA-rich algae oil, or coconut oil (placebo) for 41 days before returning to the lab to complete post-intervention testing. For this proof of concept study, the dosing strategy was chosen to ensure adequate supplementation regimens, as longer study durations and higher doses appear to induce clearer performance benefits (4). Before and after the supplementation period a blood sample was drawn from each participant’s antecubital vein and stored ready for RBC Omega-3 fatty acid analysis (see below). A range of measures were taken during a submaximal exercise step test and 24 km TT, initially to establish baseline and again post-intervention. Heart rate was measured using a HR monitor (FT1, Polar, Finland). Oxygen consumption and RER were both measured using Vyntus gas analysis equipment in the program sensory suite (Vyntus, CareFusion, Germany). This equipment was calibrated using two gas mixtures of known concentrations, as well as volume using a 3-liter syringe (Micro medical 3 liter syringe, CareFusion, Germany). RPE was measured using the 6-20-point Borg scale (18).

### Submaximal exercise step test

This test was performed on an electronically braked ergometer (Excalibur sport, Lode, Netherlands) set in hyperbolic mode. As the submaximal exercise started with very light resistance, participants were not required to do a warm-up beforehand. Participants cycled for 5 min at 100 Watts, followed by two subsequent 5-min bouts at 150 Watts and 200 Watts. Measures were taken in the last 30 s of every 5-min stage.

### Performance test (24 km TT)

The submaximal exercise was followed by a short 5 min break before performing the TT; the submaximal exercise acting as a warm-up for the more demanding TT. Similar time-trials have been used previously (19–21). Given the heterogeneity of the participants and their primary sporting disciplines there is no ideal test. The distance/time was chosen based on previous publications and the desire to strike a balance between being too short to reveal any benefits of the supplements, and being very prolonged which requires a much lower intensity where psychology/motivation become increasingly influential and metabolism less so. The same bike was used as in the submaximal exercise step test; however, it was set in linear mode. To set resistance, to suit each participant’s fitness and riding style throughout the TT, an alpha value was calculated ((0.75*Watt max)/ (rpm*rpm)) based on how the individual performed in the submaximal exercise test. The participant was then informed that resistance on the pedals would remain constant throughout this 24 km TT and requested to complete the TT as quickly as possible. Participants had any music turned off, displays for cadence, HR and oxygen consumption taken out of sight and they were informed that the investigators would not be able to talk to them throughout. Participants were informed by visual cues when 25, 50, 75 and 100% of the distance had been completed.

### Experimental standardization

Before arriving at the lab, each participant was asked to complete a food diary (3 days prior), abstain from alcohol consumption, maintain their habitual caffeine intake, and arrive in a 3-h fasted state. These control procedures were then repeated 3 days prior to the post-supplementation exercise testing. The food diaries showed compliance to the request to maintain habitual diet. Throughout the TT, room temperature was maintained at 18°C; participants were also instructed that they could consume water *ad libitum*. After this point participants were instructed to maintain their training regimes and dietary intake. Finally, pre-supplementation and post-supplementation testing were conducted on the same day of the week and same time of day.

### Supplementation

Participants on the EPA-rich condition were taking 1.8 g of EPA and 1.2 g of DHA per day. Those assigned to the DHA-rich condition were consuming 2 g of DHA and 1 g of EPA per day. Finally, the coconut oil (placebo) condition was matched at 3 g of fat per day, but only 60 mg of poly-unsaturated fatty acids a day. The DHA-rich (Omega life Vegan algae oil, Omega-3 s in triacylglyceride form) and EPA-rich (Omega life Classic, Omega-3 s in re-esterified TAG form) capsules were provided by Doetsch Grether AG, and the coconut oil placebo (coconut oil capsules) was provided by Krauterhaus Sanct Bernhard.

Participants were instructed to consume half of the supplements with breakfast and the remaining half with dinner. Compliance to this dose was monitored using returned capsule counts as well as RBC fatty acid analysis.

### Omega-3 index (RBC fatty acid analysis)

Six-seven milliliters of blood was drawn from each participant into an EDTA coated tube via venepuncture of the antecubital vein. This was then centrifuged for 10 min at 4°C and 1,560 g. One and a half mL of RBCs were then aspirated into an Eppendorf tube and labeled. The Eppendorf tubes were then stored in a freezer at −80°C for later analysis. RBC fatty acid composition was analyzed by gas chromatography with flame ionization detection at OmegaQuant Analytics (22). Fatty acid composition was expressed as a percent of total identified fatty acids.

### Statistical analysis

All data are presented in a pre to post format as Mean ± SD. A linear mixed model with three fixed variables (Condition {EPA-rich, Placebo, DHA-rich}, Visit {Baseline, Post-supplementation} & submaximal exercise power output {100 W, 150 W, 200 W}) and one cluster variable (Participant) were used to assess submaximal exercise data. Whereas a linear mixed model with two fixed variables (Condition, Visit) and one cluster variable (Participant) was used to assess TT data. Main effects and interactions were followed by *post hoc* analysis with a Bonferroni correction. All variables were assessed for normality using skewness, kurtosis and shapiro–wilk before undergoing mixed model analysis. Spearman’s correlation coefficient was used to assess strength and direction of correlations. The significance value was set at *p* ≤ 0.05. All statistical analyses were performed in Jamovi (23).

## Results

### Supplementation compliance

Based on returned capsule count mean compliance to the supplementation period for the EPA-rich group was 91.3 ± 12.1%, 92.3 ± 19.0% for the placebo group and 93.8 ± 14.2% for the DHA-rich group (non-signficant).

### Omega-3 index

**Figure 1:**
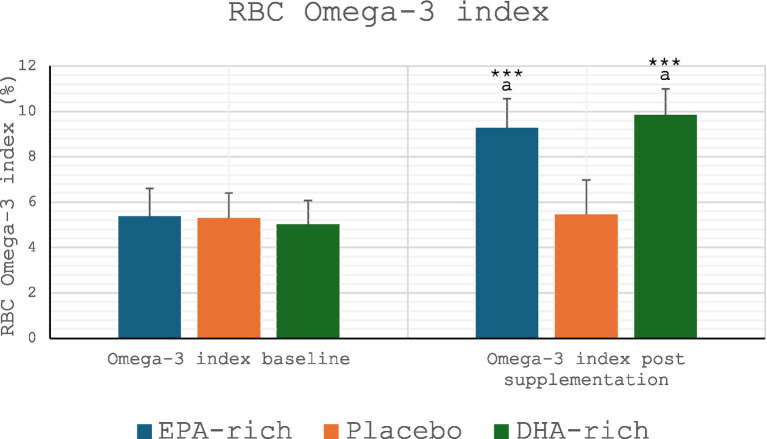
Omega-3 index: all values are Mean ± SD. *** denotes a significant difference from baseline *p* ≤ 0.001, ^a^ denotes a significant difference from placebo *p* ≤ 0.01.

There were no significant differences in participants’ Omega-3 index at baseline, i.e., pre-supplementation. After 6 weeks of supplementation the Omega-3 index increased in both the EPA-rich and DHA-rich supplemented groups (*p* ≤ 0.001) but not the control group (Figure 1). The increases were such that there was a statistically significant difference between the two experimental conditions and placebo at the end of the supplementation period (*p* < 0.01). In addition, the Omega-6 content of cell membranes decreased in the both the EPA-rich and DHA-rich supplemented groups (*p* ≤ 0.001) but not the control group (see Supplementary Figure S1).

## Submaximal data

### Submaximal HR

**Figure 2:**
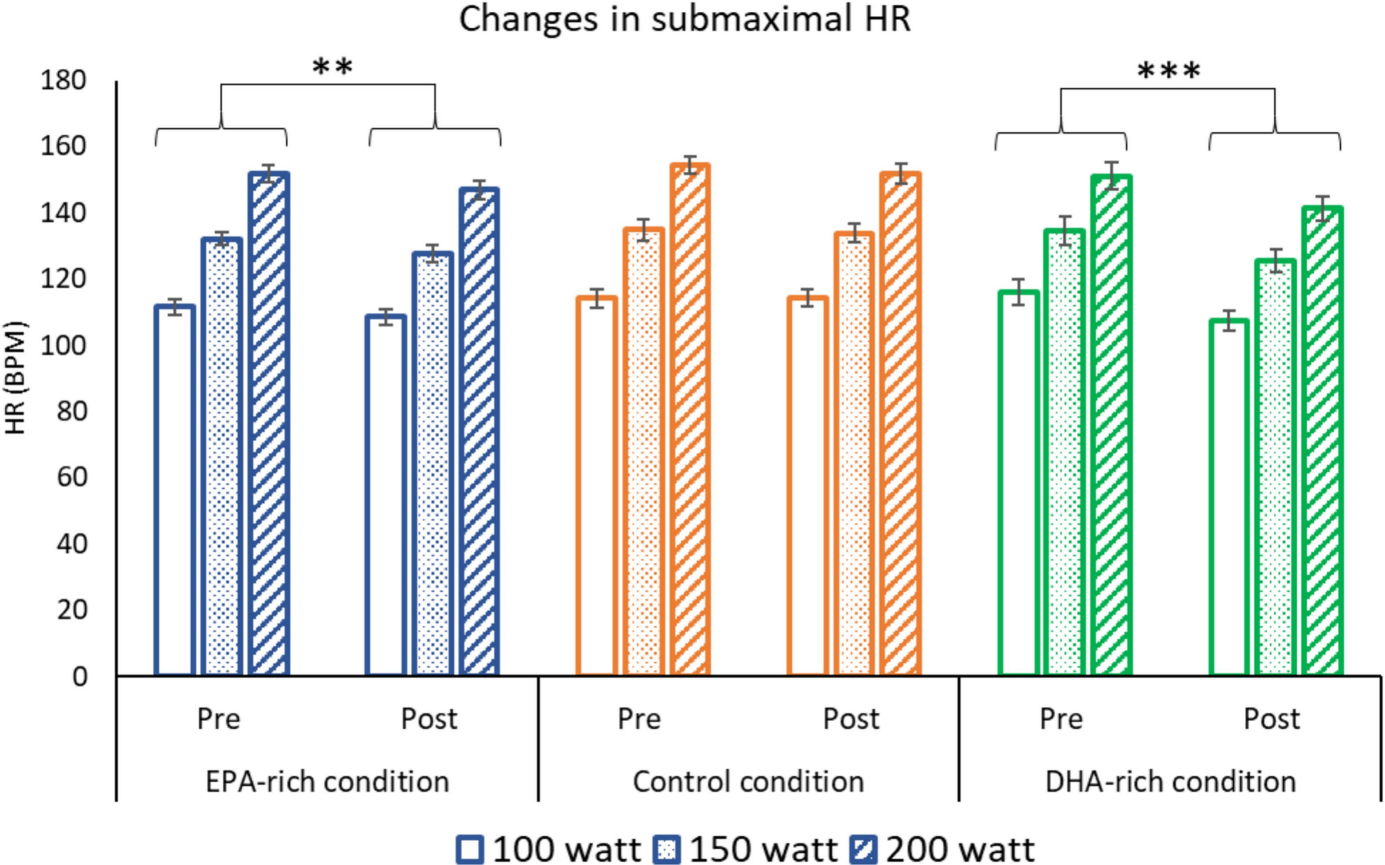
The effect of 6 weeks of EPA-rich (*n* = 18), DHA-rich (*n* = 20) or coconut oil (*n* = 17) supplementation on submaximal HR. Values are Mean ± SD. ** *p* ≤ 0.01, *** *p* ≤ 0.001 denotes differences from baseline. The main effect of intensity (200 > 150 > 100 W) is not shown on the figure (*p* < 0.001).

In all three groups, submaximal HR increased along with the intensity levels (100, 150, and 200 Watts) both at pre-and post-supplementation. However, both EPA-rich and DHA-rich supplementation lowered exercising HR, with a mean change over the three workloads of ∆ = −4 (*p* = 0.005) for EPA-rich and ∆ = −9 (*p* ≤ 0.001) for DHA-rich oils, after 6 weeks of receiving the respective oils. In contrast, no statistically significant change was observed in the placebo group from pre-to post-supplementation (Figure 2).

### Submaximal RPE

**Figure 3:**
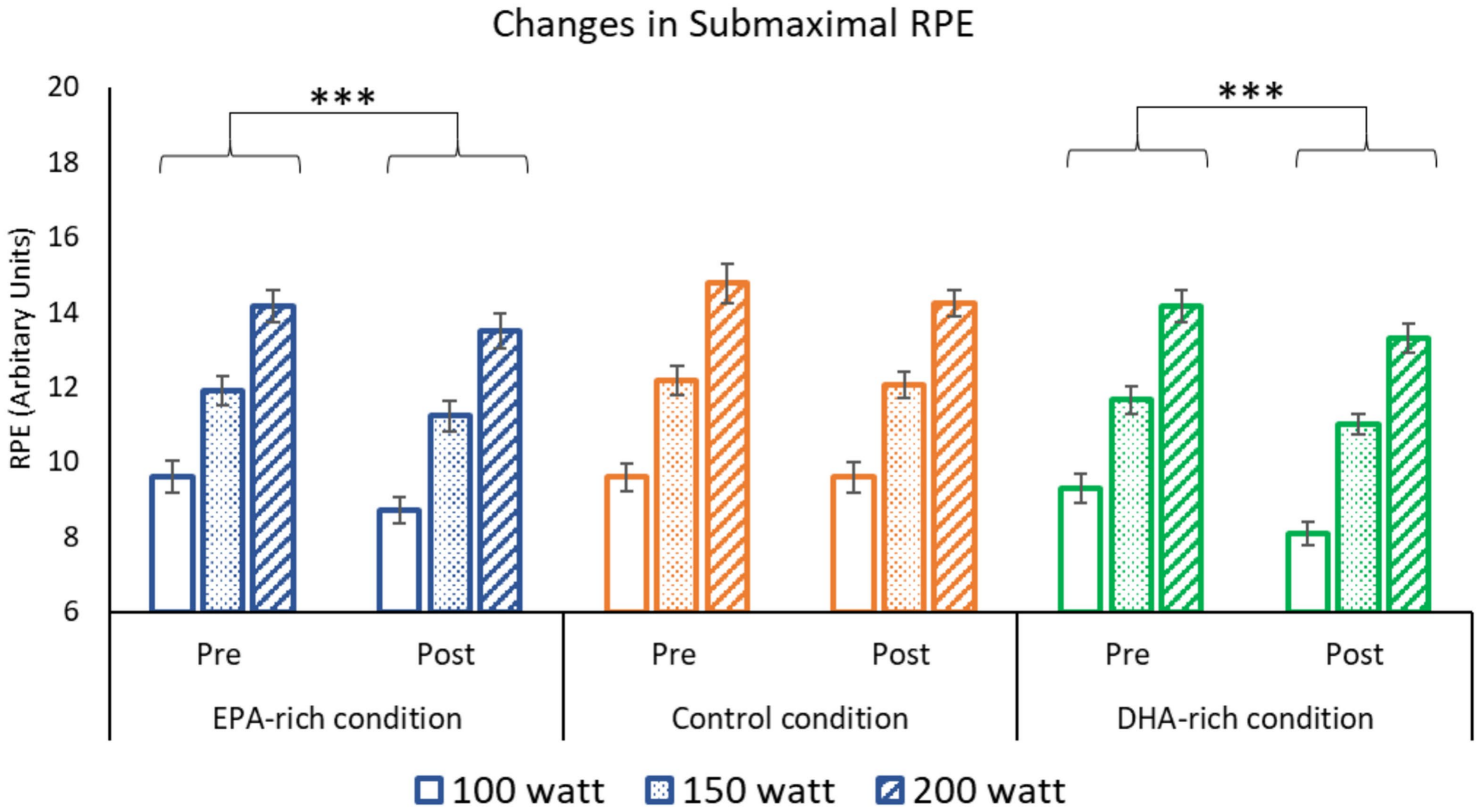
The effect of 6 weeks of EPA-rich (*n* = 18), DHA-rich (*n* = 20) or coconut oil (*n* = 17) supplementation on Submaximal RPE. Values are Mean ± SD. *** denotes difference from baseline *p* ≤ 0.001. The main effect of intensity (200 > 150 > 100 W) is not shown on the figure (*p* < 0.001).

While the submaximal RPE increased in all three groups along with the intensity levels from 100 to 200 Watts, the submaximal RPE decreased from pre-to post-supplementation in the EPA-rich oil and DHA-rich oil groups (*p* < 0.001), but not the placebo group (Figure 3).

### Submaximal RER

**Figure 4:**
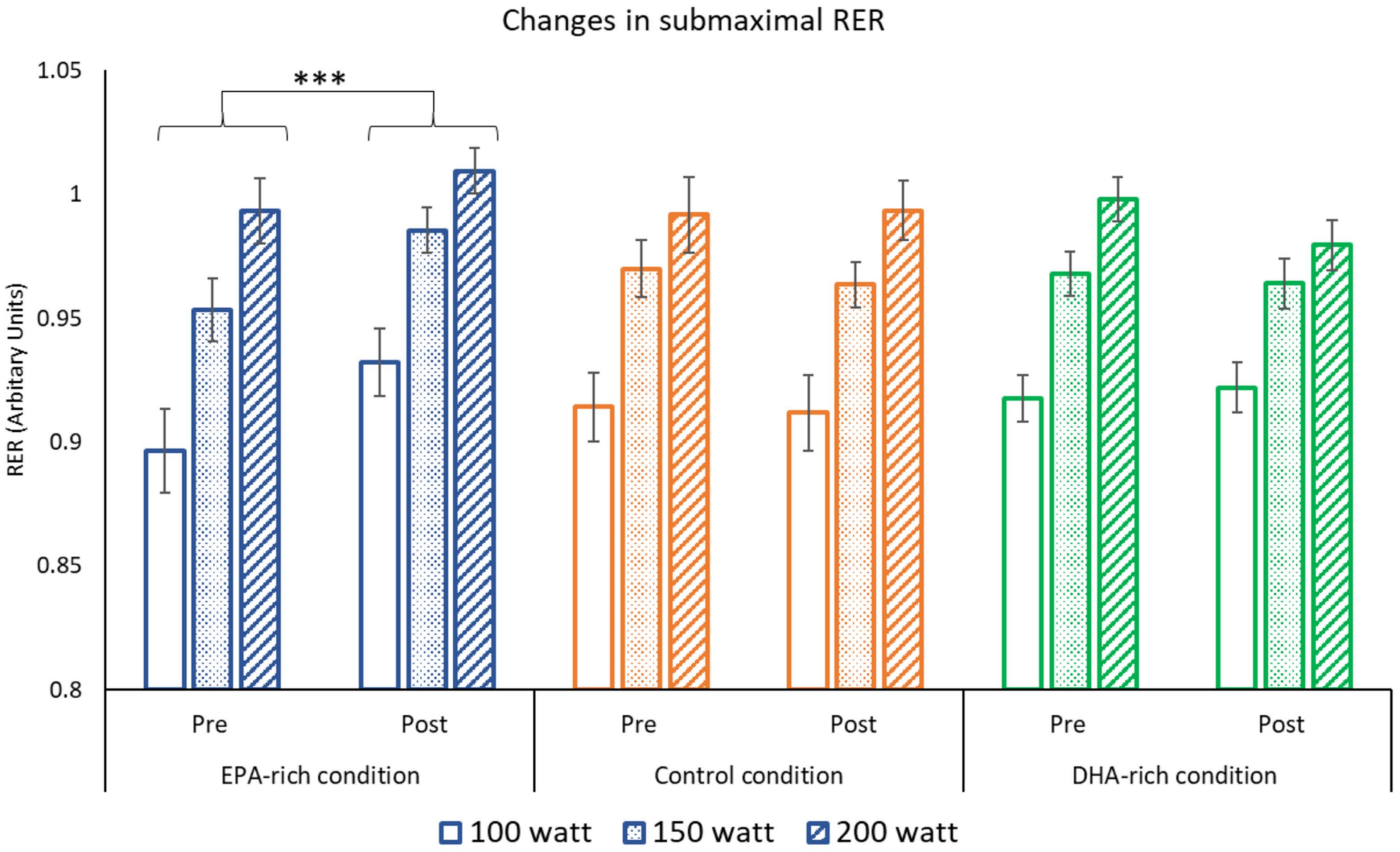
The effect of 6 weeks of EPA-rich (*n* = 18), DHA-rich (*n* = 20) or coconut oil (*n* = 17) supplementation on submaximal RER. Values are Mean ± SD. *** denotes difference from baseline *p* ≤ 0.001. The main effect of intensity (200 > 150 > 100 W) is not shown on the figure (*p* < 0.001).

The submaximal RER increased as intensity increased from 100 to 200 Watts for all three groups (All *p* ≤ 0.001). The submaximal RER also increased in the EPA-rich fish oil group from pre-to post-supplementation (*p* ≤ 0.001). While no change was observed pre-to post-supplementation in the groups that received the DHA-rich or the coconut oil control (Figure 4).

### Correlations with Omega-3 index

#### Change in submaximal HR: mean of the three intensities (100, 150, 200 W)

**Figure 5:**
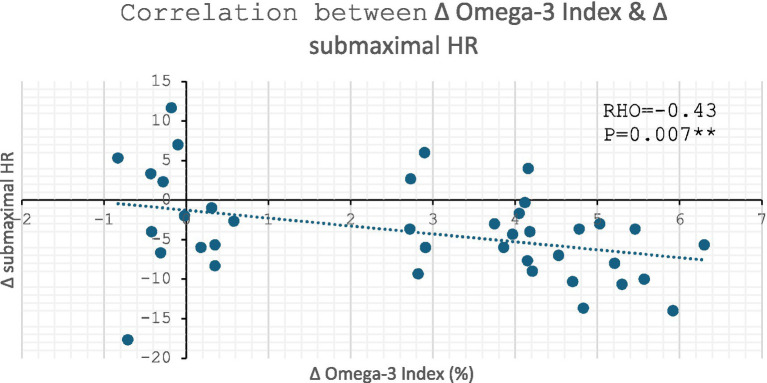
Correlation between change in Omega-3 Index vs. change in submaximal HR: ** denotes *p* ≤ 0.01.

Across all participants, when assessing the association between Omega-3 index and submaximal HR, there was a significant moderate inverse correlation between the change in Omega-3 index and the change in submaximal HR of −0.43 (*p* = 0.007, Figure 5).

There was no significant correlation between change in % of EPA in total RBC FA and change in HR (RHO = −0.23, *p* = 0.166). However, there was a moderate inverse correlation between change in % of DHA in total RBC FA and change in HR (RHO = −0.46, *p* = 0.003). Both figures are supplied in Supplementary Figures S2, S3.

#### Change in submaximal RPE

**Figure 6:**
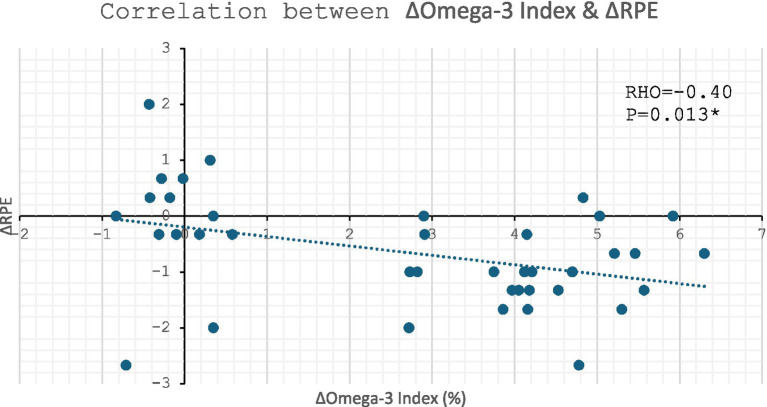
Correlation between change in Omega-3 Index vs. change in submaximal RPE: * denotes *p* ≤ 0.05.

Across all participants, there was also a significant inverse correlation between change in Omega-3 index and change in submaximal RPE (*p* = 0.013, Figure 6). The strength of −0.40 suggest this correlation to be of moderate strength.

A borderline significant inverse correlation was observed between change in DHA and change in RPE (RHO = −0.30, *p* = 0.06). However, there was a significant inverse correlation between change in EPA and change in RPE (RHO = −0.34, *p* = 0.035). Both figures are supplied in Supplementary Figures S4, S5.

### TT – time to completion

**Figure 7:**
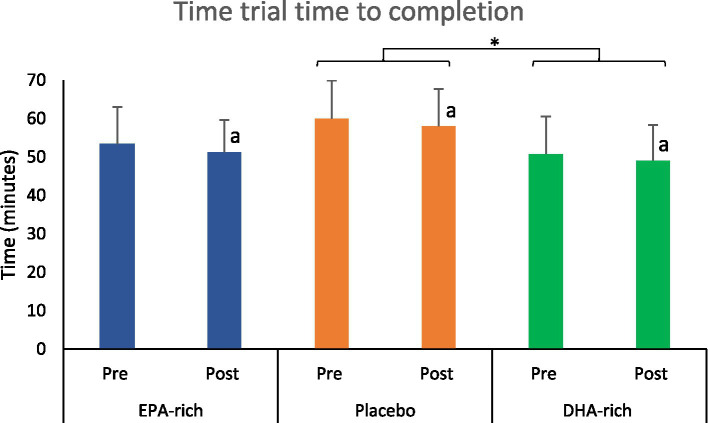
The effect of 6 weeks of EPA-rich (*n* = 18), DHA-rich (*n* = 20) or coconut oil (*n* = 17) supplementation on 24 km TT performance. Values are Mean ± SD. Symbol ^a^ indicates a significant main effect of visit. * denotes significant main effect for Condition *p* ≤ 0.05.

There was significant main effect for visit (*p* ≤ 0.001, *F* = 11.03) in time to complete the 24 km TT; participants were quicker post-supplementation compared to pre-supplementation across all intervention groups (*p* ≤ 0.001, Figure 7). There was a significant main effect for Condition (*p* = 0.012, *F* = 4.80) in time to complete the TT; further analysis revealed that the DHA-rich group was significantly quicker than the coconut oil control group (*p* = 0.014) regardless of visit. No significant interaction was found for Condition*Visit (*p* = 0.761, *F* = 0.27) in time to complete the 24 km TT (Figure 7).

### Other TT measures

TT power output, V̇O_2_, HR & RER have not been presented as they showed no meaningful changes.

## Discussion

### Main findings

Six weeks of supplementation with either EPA-rich or DHA-rich Omega-3 fatty acids increased the Omega-3 index, from levels below 6% pre-intervention to values in excess of 8% post-intervention.

The supplementation also resulted in several alterations to submaximal exercise physiology at three different intensities (100, 150 & 200 Watts). Both EPA-rich and DHA-rich supplementation decreased HR and RPE during submaximal exercise, whereas no changes were observed in the control group. Furthermore, EPA-rich fish oil increased RER at submaximal intensities with no change noted in the DHA-rich and control groups. Significant negative correlations were observed between change in Omega-3 index and change in HR and RPE. Although 24 km TT performance improved, there were no significant differences between the gains seen in the three conditions.

### Integration with current knowledge

The increase of the Omega-3 index by more than 2% may well be of physiological relevance, because previous research has suggested that an Omega-3 index in excess of 8% is recommended for athletes and provides the greatest cardioprotective effects (8). The increase in Omega-3 index reported here is comparable in magnitude to the 2-3 g/day supplementation publications summarized by Dempsey et al. (24). Incorporation of DHA into cardiac muscle is similar or even superior to incorporation into RBCs (25, 26). In the absence of cardiac and skeletal muscle biopsies, as a surrogate the incorporation of EPA and DHA into participants RBCs not only indicates compliance to the supplementation protocol but suggests that a similar or greater level of incorporation occurred at the cardiac and skeletal muscle (5).

Decreased resting HR (27, 28) and submaximal exercising HR following a period of DHA-rich supplementation is well documented (2, 29, 30). In contrast, others have found no effect on exercising HR using EPA-rich supplements (31) or supplements with almost equal doses of EPA and DHA (2,224 mg EPA + 2,208 mg DHA per day) (32). The current study’s results reveal decreased exercising HR in both DHA-rich and EPA-rich groups. Correlation data suggests that only the change in the % of DHA in total RBC FA correlated negatively with change in HR (Supplementary Figure S3), so the change in HR in the EPA-rich group might be a consequence of the 1.2 g/day DHA present in the EPA-rich condition. DHA incorporation into cardiac muscle could lower sympathetic activity and improve left ventricular function (33). However, in a study investigating the effects of Omega-3 supplementation on patients with heart transplants, the same effect was observed (34). This proves that Omega-3 fatty acids can lower HR regardless of neural stimulation. Furthermore, animal studies suggest Omega-3 supplements can slow the pacemaker current in rabbit sino-atrial nodal cells (35). In addition, research by Kang (36) showed that Omega-3 fatty acids do not need to be incorporated into the plasma membrane to lower HR. Interestingly, the same authors found that only EPA and not DHA could increase the strength of the action potential required to stimulate a cardiac myocyte. This might imply that EPA could be responsible for this decrease in HR, however the majority of reports favor DHA as the main mediator of the change in HR. Further investigation found that both EPA and DHA had a short inhibitory effect on sodium channels responsible for cardiac contraction (36). This highlights how Omega-3 fatty acids are capable of inducing lower HR at rest and during exercise.

This is the first study to show that submaximal exercise RPE decreased post-supplementation with both EPA-rich and DHA-rich supplementation in endurance trained individuals. Previous work has shown EPA-rich fish oil can lower RPE in untrained individuals (13, 37), although one such study found no benefit (31). In contrast, Peoples et al. (2) did not observe changes in RPE, but this might be explained by their well-trained participants and/or the DHA-rich supplement. The dosage of EPA in some previous work (13, 37) was similar to the dosage of EPA in the DHA-rich group of our study and could explain why similar effects were seen in both groups. Correlation data from the current study lends some support to the idea that EPA is driving the change in RPE, as only the change in % of EPA in total RBC FA was negatively correlated with change in RPE (Supplementary Figure S4), although it is worth noting this was only a weak correlation. It is possible that EPA might be responsible for this observation, however the mechanisms of this remain unclear. Nevertheless, this finding does indicate that exercise efficiency was increased in both Omega-3 groups. Omega-3 fatty acids have been shown to increase exercise efficiency in a few other studies (2, 13). While previous studies have often attributed improvements in exercise efficiency to a reduced oxygen requirement, this effect was not observed in the present study.

Current research presents inconsistent findings regarding the effects of EPA-rich Omega-3 fatty acid supplementation on submaximal RER and substrate oxidation. One study has reported a decrease in RER and an increase in fat metabolism in elderly females (38). Whereas other studies have found that RER increased in healthy young males (39). The findings of the current study align with those reported by Pearson et al. (39). While EPA-rich supplementation may influence RER differently based on factors such as age or sex, further research is needed to substantiate these potential variations.

This is not the first study investigating the effect of Omega-3 fatty acids to uncover positive changes in submaximal exercise, but no meaningful change in endurance performance (2, 13, 40). Other studies have also found no change in endurance performance after supplementation with Omega-3 fatty acids (6, 9, 10, 12, 31, 41–44), however, this is the first study to find no additional change in performance compared with the use of a true placebo in conjunction with an appropriate performance measure, i.e., TT.

### Strengths and limitations

Althouth the current study has some limitations, there are notable positives that are not always present in the literature. The focus on male participants limits the generalization of the results, particularly given the known differences in metabolism between sexes. The use of a familiarization session is important, although it is possible that one familiarization session might not be enough to prevent an order effect. The double blind approach employed in the current study should also be adopted in future studies to limit bias. Furthermore, this study utilized a placebo which was neither high in Omega-3 nor Omega-6, so reduced the risk of altering Omega 3:6 absorption and/or cell membrane incorporation. Finally, this study shows a clear increase in the Omega-3 index, in both experimental conditions, but not the placebo. The data illustrates that 6 weeks of supplementation is sufficient to elevate the Omega-3 index above the recommended threshold of 8%.

This is the first study of its kind to investigate the effects of both EPA-rich and DHA-rich Omega-3 fatty acids side by side and observe changes in submaximal data but not performance compared with placebo. This study continues to add evidence to the paradox that is currently observed in Omega-3 performance data. Having demonstrated a decreased submaximal RPE & HR in both groups it is unclear why the performance benefit seen in the current TT in either experimental group was not different to the improved performance in the placebo condition. It is possible participants learnt how to perform the TT more efficiently, despite undergoing a familiarization session; a second or third familiarization session could be added in future studies. It is also possible that participants changed their training over the 6-week supplementation period, this training may have made physiological adaptations that would therefore improve their TT performance and mask potential effects of EPA and DHA. To monitor this, future studies should collect training diaries. Another possible explanation is that the null finding for performance is a type 2 error. However, the number of participants in the current study is similar or more than the majority of the published research on Omega-3 and performance. Moreover, the *p* value for the TT interaction was far from being statistically significant (*p* = 0.76), indicating that it was not close to a performance benefit (45). Finally, it is possible the generous dose of EPA and DHA in both conditions might provide sufficient amounts of either Omega-3 FA to induce an effect, thus preventing comparison of their efficacy to induce physiological changes during submaximal exercise. Future research could investigate the effects of pure EPA and DHA to improve understanding in this area.

The Psychobiological model suggest that endurance performance is regulated by the perception of effort (46). Due to a decreased RPE in both supplemented groups at submaximal intensities it would be a reasonable prediction to expect endurance performance to improve during a TT. Unfortunately, the current study did not measure RPE through the TT therefore making it difficult to conclude if participants did find the TT less effort. Furthermore, a lower HR at submaximal intensities suggests improved cardiac efficiency. However, if the cardiac system was not a limiting factor in performance of the 24 km TT then the apparent disconnect between lower HR and lack of performance gains is perhaps not surprising. For example, the TT is very leg dominated; if leg strength or leg muscle metabolism becomes the limiting factor to performance, then improvement of cardiac efficiency would have little to no bearing on TT performance. For a more in depth discussion of the possible mechanisms for Omega-3-induced performance gains readers are referred to the recent paper by Jager et al. (45).

In conclusion, a 6-week supplementation with either EPA-rich or DHA-rich Omega-3 fatty acids is sufficient to enhance the Omega-3 index to a physiologically meaningful level. Furthermore, both the EPA-rich and DHA-rich supplementation can improve cardiac efficiency, as evidenced by reduced HR during submaximal exercise intensities. A potential differential impact of EPA and DHA on substrate utilization during exercise warrants further exploration in future research. While Omega-3 supplementation may enhance certain aspects of submaximal exercise physiology, it does not translate to performance improvements in this specific endurance exercise context. To ensure the generalizability of these findings and to understand the impact of sex on the metabolic processes being studied, future studies should ideally include both male and female participants.

## Data Availability

The raw data supporting the conclusions of this article will be made available by the authors, without undue reservation.
