# Supplementary material for: Six weeks of either EPA-rich or DHA-rich Omega-3 supplementation alters submaximal exercise physiology in endurance trained male amateurs
Source: Front Nutr. 2025 Aug 26;12:1588421. doi: 10.3389/fnut.2025.1588421 (PMC12417169; doi:10.3389/fnut.2025.1588421)
Supplement: Supplementary file 1 [file Image_1.PDF]

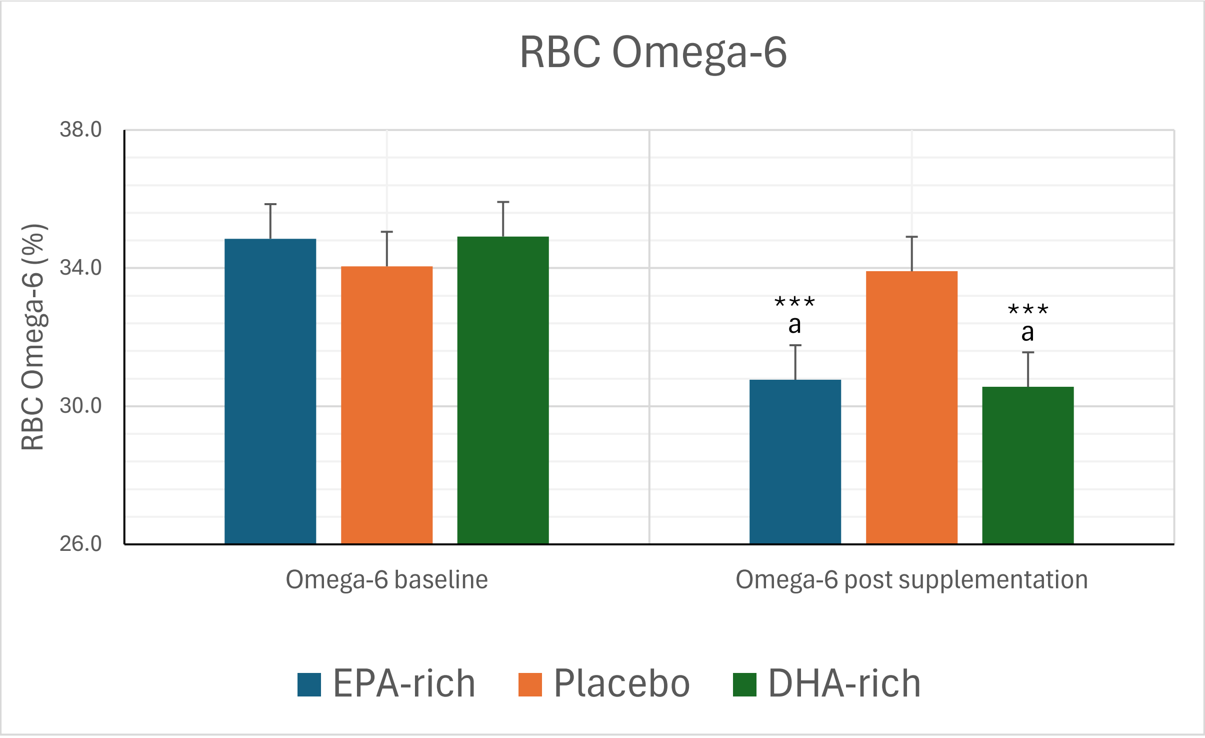

Supplementary Figure S1, RBC membrane Omega-6: All values are Mean ± SD. *** denotes a significant difference from baseline P≤0.001, a denotes a significant difference from placebo P≤0.001.

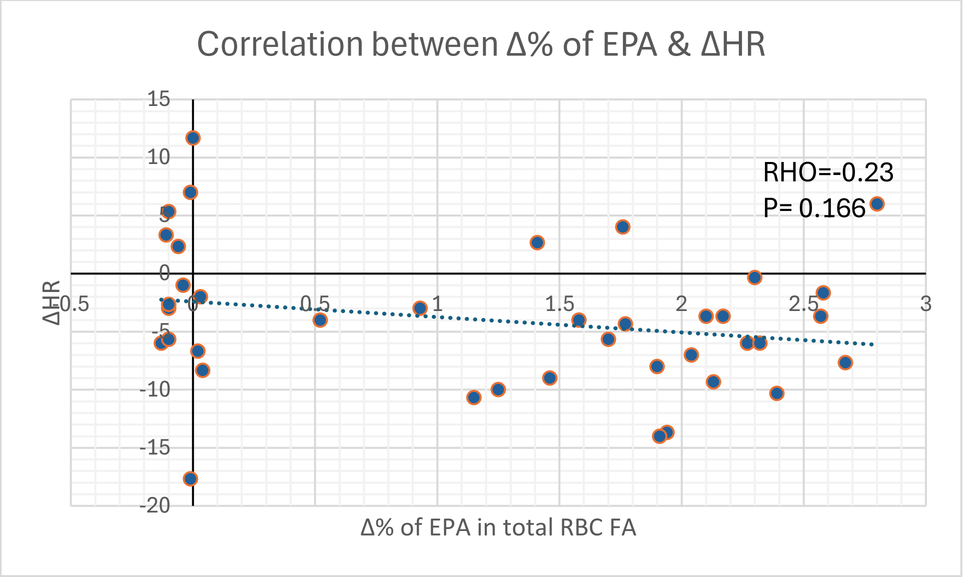

Supplementary Figure S2, Correlation between Δ % of EPA and Δ submaximal HR.

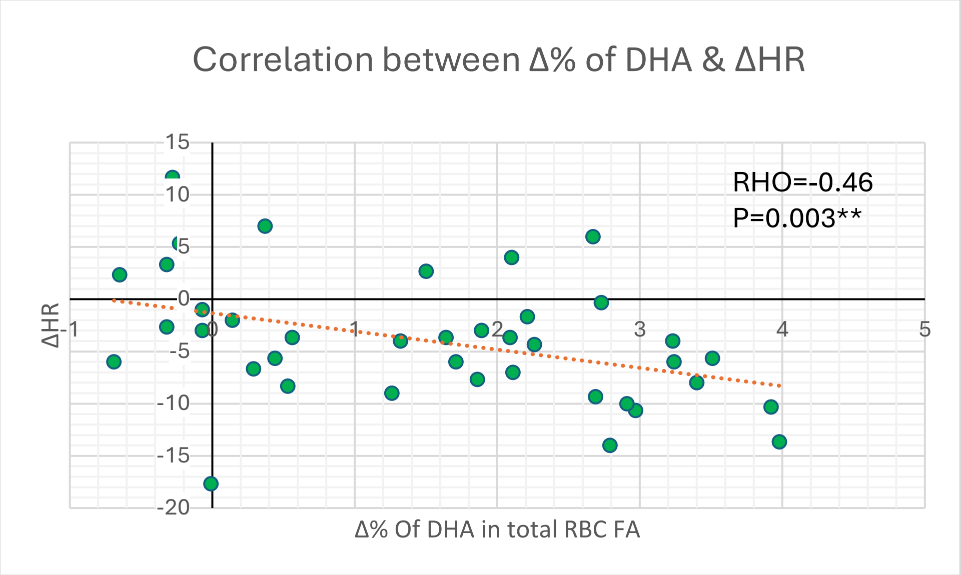

Supplementary Figure S3, Correlation between Δ % of DHA and Δ submaximal HR: ** denotes p≤0.01

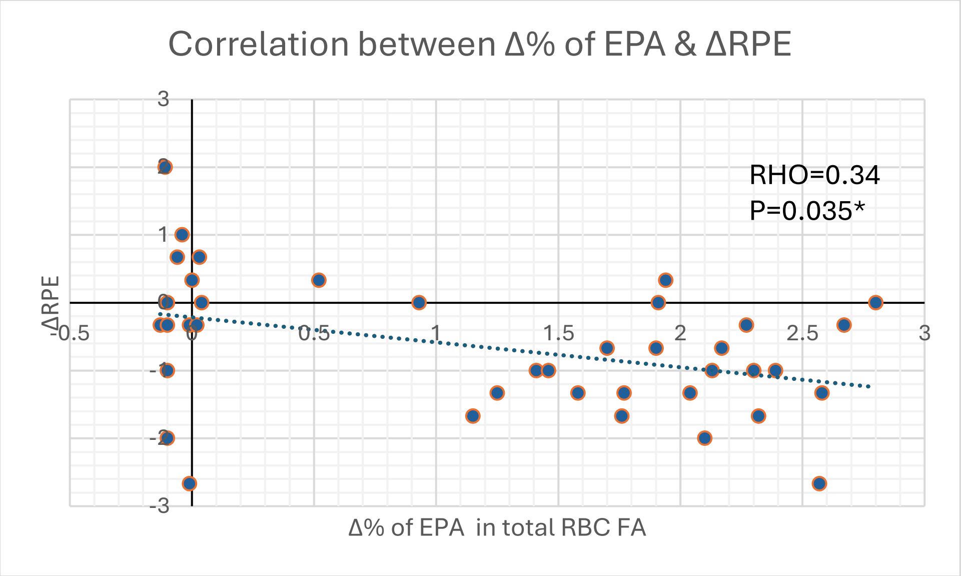

Supplementary Figure S4, Correlation between Δ % of EPA and Δ RPE: * denotes p≤0.05

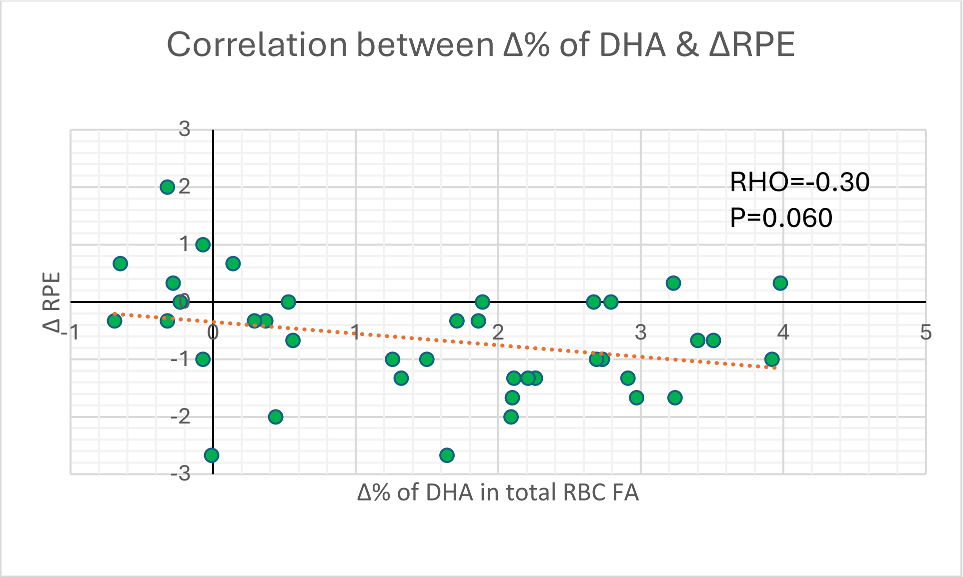

Supplementary Figure S5, Correlation between Δ % of DHA and Δ RPE.
